# Tl_2_Mo_9_Se_11_

**DOI:** 10.1107/S1600536810026541

**Published:** 2010-07-10

**Authors:** Patrick Gougeon, Philippe Gall, Michel Potel

**Affiliations:** a Sciences Chimiques de Rennes, CSM-INSA, UMR CNRS No. 6226, Université de Rennes I, Avenue du Général Leclerc, 35042 Rennes CEDEX, France

## Abstract

The structure of Tl_2_Mo_9_Se_11_, dithallium nona­molybdenum undeca­selenide, is isotypic with Tl_2_Mo_9_S_11_ [Potel *et al.* (1980 ▶). *Acta Cryst*. B**36**, 1319–1322]. The structural set-up is characterized by a mixture of Mo_6_Se^*i*^_8_Se^*a*^_6_ and Mo_12_Se^*i*^_14_Se^*a*^_6_ cluster units in a 1:1 ratio. Both components are inter­connected through inter­unit Mo—Se bonds. The cluster units are centered at Wyckoff positions 3*a* and 3*b* (point-group symmetry 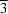.). The two Tl^I^ atoms are situated in the voids of the three-dimensional arrangement. Two of the five independent Se atoms and the Tl atoms lie on sites with 3. symmetry (Wyckoff site 6*c*).

## Related literature

For the crystal structure of Tl_2_Mo_9_S_11_, see: Potel *et al.* (1980 ▶). For details of the *i*- and *a*-type ligand notation, see: Schäfer & von Schnering (1964 ▶). Ionic radii were compiled by Shannon (1976 ▶). For background to the extinction correction, see: Becker & Coppens (1974 ▶).

## Experimental

### 

#### Crystal data

Tl_2_Mo_9_Se_11_
*M* *_r_* = 2140.8
Trigonal, 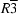

*a* = 9.6212 (1) Å
*c* = 36.3316 (7) Å
*V* = 2912.55 (7) Å^3^
*Z* = 6
Mo *K*α radiation
μ = 42.73 mm^−1^
*T* = 293 K
0.13 × 0.12 × 0.11 mm

#### Data collection

Nonius KappaCCD diffractometer
Absorption correction: multi-scan (*PLATON*; Spek, 2009 ▶) *T*_min_ = 0.033, *T*_max_ = 0.108
21097 measured reflections
4097 independent reflections
3156 reflections with *I* > 2σ(*I*)
*R*_int_ = 0.087

#### Refinement

*R*[*F* ^2^ > 2σ(*F* ^2^)] = 0.043
*wR*(*F* ^2^) = 0.092
*S* = 1.37
4097 reflections
69 parameters
Δρ_max_ = 3.92 e Å^−3^
Δρ_min_ = −2.89 e Å^−3^

### 

Data collection: *COLLECT* (Nonius, 1998 ▶); cell refinement: *COLLECT*; data reduction: *EVALCCD* (Duisenberg *et al.*, 2003 ▶); program(s) used to solve structure: *SIR97* (Altomare *et al.*, 1999 ▶); program(s) used to refine structure: *JANA2006* (Petříček *et al.*, 2006 ▶); molecular graphics: *DIAMOND* (Brandenburg, 2001 ▶); software used to prepare material for publication: *JANA2006*.

## Supplementary Material

Crystal structure: contains datablocks global, I. DOI: 10.1107/S1600536810026541/wm2368sup1.cif

Structure factors: contains datablocks I. DOI: 10.1107/S1600536810026541/wm2368Isup2.hkl

Additional supplementary materials:  crystallographic information; 3D view; checkCIF report

## Figures and Tables

**Table 1:** Selected bond lengths (Å)

Tl1—Tl2^i^	3.5164 (7)
Tl1—Se2	4.3543 (6)
Tl1—Se2^ii^	4.3543 (7)
Tl1—Se2^iii^	4.3543 (7)
Tl1—Se3	3.5840 (6)
Tl1—Se3^ii^	3.5840 (5)
Tl1—Se3^iii^	3.5840 (8)
Tl1—Se3^iv^	3.4547 (6)
Tl1—Se3^v^	3.4547 (8)
Tl1—Se3^vi^	3.4547 (5)
Tl1—Se4^vii^	3.0737 (10)
Tl2—Se1	3.4032 (7)
Tl2—Se1^viii^	3.4032 (6)
Tl2—Se1^ix^	3.4032 (8)
Tl2—Se2^i^	3.1441 (4)
Tl2—Se2^x^	3.1441 (7)
Tl2—Se2^xi^	3.1441 (7)
Tl2—Se3^i^	4.2446 (7)
Tl2—Se3^x^	4.2446 (6)
Tl2—Se3^xi^	4.2446 (8)
Tl2—Se5	3.0483 (9)
Mo1—Mo1^xii^	2.6897 (7)
Mo1—Mo1^x^	2.7609 (6)
Mo1—Mo2^viii^	3.4239 (8)
Mo1—Se1	2.5540 (8)
Mo1—Se1^x^	2.6143 (8)
Mo1—Se1^xiii^	2.5757 (6)
Mo1—Se2	2.6247 (8)
Mo1—Se4	2.5269 (8)
Mo2—Mo2^ix^	2.6382 (5)
Mo2—Mo3	2.7397 (7)
Mo2—Mo3^viii^	2.7901 (7)
Mo2—Se1^i^	2.6597 (6)
Mo2—Se2	2.6034 (9)
Mo2—Se2^ix^	2.6124 (10)
Mo2—Se3	2.6721 (6)
Mo2—Se5	2.5129 (8)
Mo3—Mo3^ix^	2.6780 (7)
Mo3—Mo3^xiv^	2.6796 (6)
Mo3—Se2^ix^	2.5634 (8)
Mo3—Se3	2.6090 (6)
Mo3—Se3^ix^	2.6112 (6)
Mo3—Se3^xiv^	2.7025 (8)

Symmetry codes: (i) 
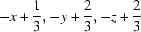
; (ii) 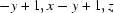; (iii) 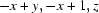; (iv) 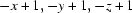; (v) 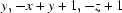; (vi) 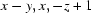; (vii) 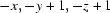; (viii) 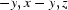; (ix) 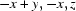; (x) 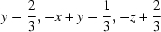; (xi) 
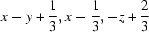
; (xii) 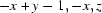; (xiii) 
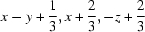
; (xiv) 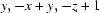.

**Fig. 1.**
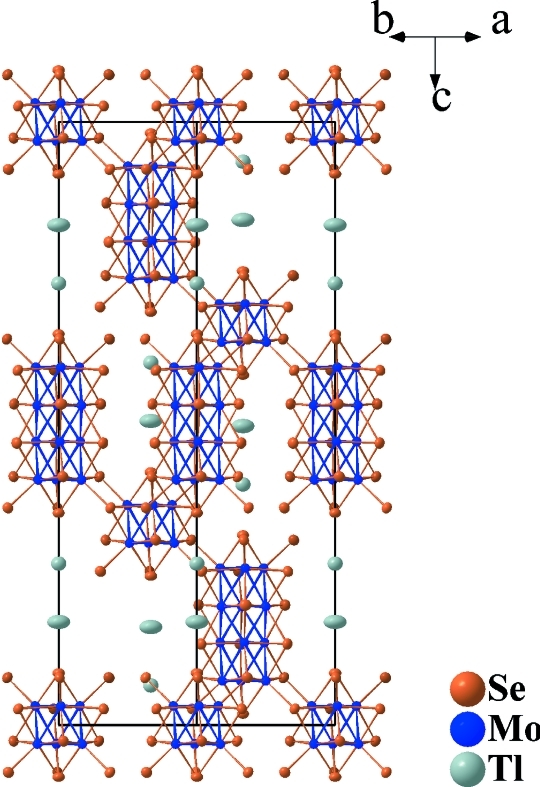
: View of Tl2Mo9Se11 along [110].

**Fig. 2.**
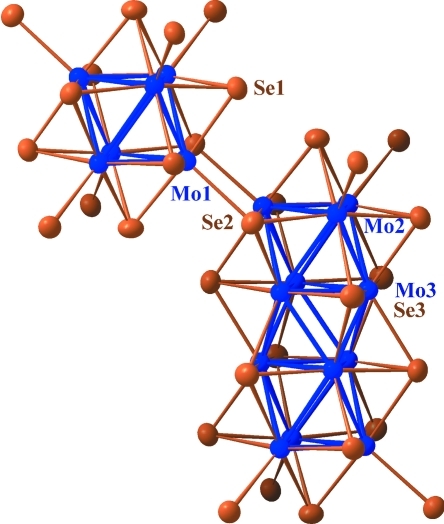
: Plot showing the atom-numbering scheme and the linkage of the Mo12Se14Se6 and Mo6Se8Se6 cluster units. Displacement ellipsoids are drawn at the 97% probability level. Symmetry codes are as in Table 1.

**Fig. 3.**
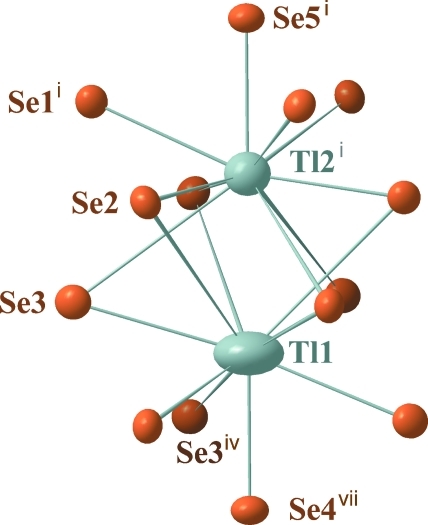
: Environment of the Tl atoms. Symmetry codes are as in Table 1.

## supplementary crystallographic information

### Comment

Potel *et al.* (1980) reported the crystal structure of Tl_2_Mo_9_S_11_ that was the first compound containing a transition metal cluster with a nuclearity higher than 9, namely the trioctahedral Mo_12_ cluster. The latter cluster, which results from the uniaxial face-sharing of three Mo_6_ octahedra, coexists with the octahedral Mo_6_ cluster in equal proportion. We present here the crystal structure of the isotypic selenide, Tl_2_Mo_9_Se_11_.

The Mo—Se framework of Tl_2_Mo_9_Se_11_ consists of an equal mixture of Mo_6_Se*^i^*_8_Se*^a^*_6_ and Mo_12_Se*^i^*_14_Se*^a^*_6_ cluster units interconnected through Mo—Se bonds (Fig.1 and 2). Details of the *i*- and *a*-type ligand notation were reported by Schäfer & von Schnering (1964). The first unit can be described as an Mo_6_ octahedron surrounded by 8 face-capping inner Se*^i^* and 6 apical Se*^a^* ligands. The Mo_12_ core of the second unit results from the face sharing of 3 octahedral Mo_6_ clusters. The Mo_12_ cluster is surrounded by 14 Se*^i^* atoms capping the faces of the bioctahedron and 6 apical Se*^a^* ligands above the ending Mo atoms. The Mo_6_Se*^i^*_8_Se*^a^*_6_ and Mo_12_Se*^i^*_14_Se*^a^*_6_ units are centered at 3*a* and 3*b* positions and have point-group symmetry 3. The Mo—Mo distances within the Mo_6_ clusters are 2.6897 (7) Å for the intra-triangle distances (distances within the Mo_3_ triangles formed by the Mo1 atoms related through the threefold axis) and 2.7609 (6) Å for the inter-triangle distances. In the sulfide, the two later values are slightly larger, *viz*. 2.693 (1) and 2.780 (5) Å. The Mo—Mo distances within the Mo_12_ clusters are 2.6382 (5) and 2.6780 (7) Å for the distances in the triangles formed by the Mo2 and Mo3 atoms, respectively. In Tl_2_Mo_9_S_11_, the corresponding distances are equal to 2.658 (1) and 2.688 (1) Å, respectively. The distances between the triangles formed by the Mo2 and Mo3 atoms are 2.7397 (7) and 2.7901 (7) Å and those between the Mo3_3_ triangles, 2.6796 (6) Å. The average Mo—Mo distance within the Mo_12_ cluster is similar in Tl_2_Mo_9_S_11_ and Tl_2_Mo_9_Se_11_ and amounts to 2.705 Å. The Se atoms bridge either one (Se1, Se2, Se4 and Se5) or two (Se3) Mo triangular faces of the clusters. Moreover, the Se1 and Se2 atoms are linked to a Mo atom of a neighboring cluster. The Mo—Se bond distances range from 2.5269 (8) to 2.6247 (8) Å within the Mo_6_Se^i^_8_Se*^a^*_6_ unit and from 2.5129 (8) to 2.7025 (8) Å within the Mo_12_Se*^i^*_14_Se*^a^*_6_ ubit. Each Mo_12_Se^i^_14_Se*^a^*_6_ unit is interconnected to 6 Mo_6_Se*^i^*_8_Se*^a^*_6_ units (and *vice versa*) *via* Mo2—Se1 bonds (respectively Mo1—Se2) to form the three-dimensional Mo—Se framework, the overall connectivity formula of which is Mo_12_Se*^i^*_8_Se*^i-a^*_6/2_Se*^a-i^*_6/2_, Mo_6_Se*^i^*_2_Se*^i-a^*_6/2_Se*^a-i^*_6/2_. It results from this arrangement that the shortest intercluster Mo1—Mo2 distance between the Mo_6_ and Mo_12_ clusters is 3.4239 (8) Å compared to 3.217 (1) in Tl_2_Mo_9_S_11_, indicating only weak metal-metal interaction. The Tl atoms occupy large voids delimited by four Mo_6_Se*^i^*_8_Se*^a^*_6_ units and four Mo_12_Se*^i^*_14_Se*^a^*_6_ units. The Tl1 and Tl2 cations have a very similar environment which consists of seven Se atoms as nearest neighbors with Tl—Se distances in the ranges 3.0737 (10) - 3.5840 (6) Å and 3.0483 (9) - 3.4032 (6) Å for the Tl1 and Tl2 sites, respectively. These seven Se atoms form a monocapped octahedron compressed along the threefold axis. Three additional Se atoms at 4.3543 (6) and 4.2446 (6) Å from Tl1 and Tl2, respectively, are also observed (Figure 3). The average Tl—Se values are 3.72 and 3.54 Å for the Tl1 and Tl2 sites, respectively. Values of 3.57 and 3.68 Å are expected from the sum of the ionic radii for Se^2-^ and Tl^+^ for coordination numbers 8 and 12 (Shannon, 1976).

### Experimental

Single crystals of Tl_2_Mo_9_Se_11_ were prepared from a mixture of MoSe_2_, TlSe and Mo with the nominal composition Tl_2_Mo_12_Se_14_. Before use, Mo powder was reduced under H_2_ flowing gas at 1273 K during ten hours in order to eliminate any trace of oxygen. The binaries MoSe_2_ and TlSe were obtained by heating stoichiometric mixtures of the elements in sealed evacuated silica tubes during about 2 days at 1073 and 573 K, respectively. All handlings of materials were done in an argon-filled glove box. The initial mixture (*ca* 5 g) was cold pressed and loaded into a molybdenum crucible, which was sealed under a low argon pressure using an arc welding system. The charge was heated at the rate of 300 K/h up to 1773 K, the temperature which was held for 48 h, then cooled at 100 K/h down to 1373 K and finally furnace cooled.

### Refinement

The highest peak and the deepest hole in the final Fourier map are at 1.67 and 0.83 Å from Tl1 and Se5, respectively.

### Crystal data

**Table:**

Tl_2_Mo_9_Se_11_	*D*_x_ = 7.321 Mg m^−^^3^
*M**_r_* = 2140.8	Mo *K*α radiation, λ = 0.71069 Å
Trigonal, *R*3	Cell parameters from 10017 reflections
Hall symbol: -R 3	θ = 1–40.3°
*a* = 9.6212 (1) Å	µ = 42.73 mm^−^^1^
*c* = 36.3316 (7) Å	*T* = 293 K
*V* = 2912.55 (7) Å^3^	Irregular block, black
*Z* = 6	0.13 × 0.12 × 0.11 mm
*F*(000) = 5484	

### Data collection

**Table:**

Nonius KappaCCD diffractometer	4097 independent reflections
Radiation source: fine-focus sealed tube	3156 reflections with *I* > 2σ(*I*)
horizontally mounted graphite crystal	*R*_int_ = 0.087
Detector resolution: 9 pixels mm^-1^	θ_max_ = 40.2°, θ_min_ = 1.7°
φ– and ω–scans	*h* = −16→17
Absorption correction: multi-scan (*PLATON*; Spek, 2009)	*k* = −16→17
*T*_min_ = 0.033, *T*_max_ = 0.108	*l* = −58→66
21097 measured reflections	

### Refinement

**Table:**

Refinement on *F*^2^	0 constraints
*R*[*F*^2^ > 2σ(*F*^2^)] = 0.043	Weighting scheme based on measured s.u.'s *w* = 1/[σ^2^(*I*) + 0.0004*I*^2^]
*wR*(*F*^2^) = 0.092	(Δ/σ)_max_ = 0.033
*S* = 1.37	Δρ_max_ = 3.92 e Å^−^^3^
4097 reflections	Δρ_min_ = −2.89 e Å^−^^3^
69 parameters	Extinction correction: B-C type 1 Gaussian isotropic (Becker & Coppens, 1974)
0 restraints	Extinction coefficient: 2320 (40)

### Fractional atomic coordinates and isotropic or equivalent isotropic displacement parameters (Å^2^)

**Table:**

	*x*	*y*	*z*	*U*_iso_*/*U*_eq_	
Tl1	0.333333	0.666667	0.495781 (14)	0.04933 (17)	
Tl2	0	0	0.267672 (12)	0.02611 (11)	
Mo1	−0.18022 (5)	0.31787 (5)	0.364749 (12)	0.01129 (13)	
Mo2	0.16529 (5)	0.15027 (5)	0.406587 (12)	0.01120 (13)	
Mo3	0.15606 (5)	−0.00891 (5)	0.469882 (12)	0.01087 (13)	
Se1	−0.04118 (6)	0.29911 (6)	0.306626 (15)	0.01295 (17)	
Se2	0.03067 (6)	0.32633 (6)	0.411871 (15)	0.01342 (17)	
Se3	0.32150 (6)	0.30388 (6)	0.467628 (15)	0.01463 (17)	
Se4	−0.333333	0.333333	0.41962 (2)	0.01559 (19)	
Se5	0	0	0.35157 (2)	0.01474 (19)	

### Atomic displacement parameters (Å^2^)

**Table:**

	*U*^11^	*U*^22^	*U*^33^	*U*^12^	*U*^13^	*U*^23^
Tl1	0.0620 (2)	0.0620 (2)	0.0239 (2)	0.03102 (12)	0	0
Tl2	0.02704 (13)	0.02704 (13)	0.0242 (2)	0.01352 (7)	0	0
Mo1	0.01119 (17)	0.01064 (17)	0.01168 (17)	0.00518 (13)	0.00061 (13)	−0.00028 (13)
Mo2	0.01129 (17)	0.01096 (17)	0.01107 (17)	0.00535 (14)	0.00023 (13)	0.00051 (13)
Mo3	0.01081 (16)	0.01111 (16)	0.01077 (17)	0.00553 (13)	0.00040 (13)	0.00014 (13)
Se1	0.0120 (2)	0.0136 (2)	0.0140 (2)	0.00691 (17)	0.00193 (16)	0.00090 (16)
Se2	0.0145 (2)	0.0121 (2)	0.0140 (2)	0.00685 (17)	−0.00087 (17)	0.00125 (16)
Se3	0.0121 (2)	0.0135 (2)	0.0151 (2)	0.00408 (17)	0.00057 (17)	0.00032 (17)
Se4	0.0181 (2)	0.0181 (2)	0.0106 (3)	0.00905 (12)	0	0
Se5	0.0167 (2)	0.0167 (2)	0.0108 (3)	0.00835 (11)	0	0

### Geometric parameters (Å, °)

**Table:**

Tl1—Tl2^i^	3.5164 (7)	Mo1—Mo1^xiv^	2.7609 (7)
Tl1—Se2	4.3543 (6)	Mo1—Mo2^viii^	3.4239 (8)
Tl1—Se2^ii^	4.3543 (7)	Mo1—Se1	2.5540 (8)
Tl1—Se2^iii^	4.3543 (7)	Mo1—Se1^x^	2.6143 (8)
Tl1—Se3	3.5840 (6)	Mo1—Se1^xiv^	2.5757 (6)
Tl1—Se3^ii^	3.5840 (5)	Mo1—Se2	2.6247 (8)
Tl1—Se3^iii^	3.5840 (8)	Mo1—Se4	2.5269 (8)
Tl1—Se3^iv^	3.4547 (6)	Mo2—Mo2^viii^	2.6382 (6)
Tl1—Se3^v^	3.4547 (8)	Mo2—Mo2^ix^	2.6382 (5)
Tl1—Se3^vi^	3.4547 (5)	Mo2—Mo3	2.7397 (7)
Tl1—Se4^vii^	3.0737 (10)	Mo2—Mo3^viii^	2.7901 (7)
Tl2—Se1	3.4032 (7)	Mo2—Se1^i^	2.6597 (6)
Tl2—Se1^viii^	3.4032 (6)	Mo2—Se2	2.6034 (9)
Tl2—Se1^ix^	3.4032 (8)	Mo2—Se2^ix^	2.6124 (10)
Tl2—Se2^i^	3.1441 (4)	Mo2—Se3	2.6721 (6)
Tl2—Se2^x^	3.1441 (7)	Mo2—Se5	2.5129 (8)
Tl2—Se2^xi^	3.1441 (7)	Mo3—Mo3^viii^	2.6780 (9)
Tl2—Se3^i^	4.2446 (7)	Mo3—Mo3^ix^	2.6780 (7)
Tl2—Se3^x^	4.2446 (6)	Mo3—Mo3^xv^	2.6796 (6)
Tl2—Se3^xi^	4.2446 (8)	Mo3—Mo3^vi^	2.6796 (7)
Tl2—Se5	3.0483 (9)	Mo3—Se2^ix^	2.5634 (8)
Mo1—Mo1^xii^	2.6897 (9)	Mo3—Se3	2.6090 (6)
Mo1—Mo1^xiii^	2.6897 (7)	Mo3—Se3^ix^	2.6112 (6)
Mo1—Mo1^x^	2.7609 (6)	Mo3—Se3^xv^	2.7025 (8)
			
Mo1^xii^—Mo1—Mo1^xiii^	60.000 (18)	Mo2—Mo3—Mo3^ix^	90.11 (2)
Mo1^xii^—Mo1—Mo1^x^	90	Mo2—Mo3—Mo3^xv^	147.49 (3)
Mo1^xii^—Mo1—Mo1^xiv^	60.85 (2)	Mo2—Mo3—Mo3^vi^	111.83 (2)
Mo1^xiii^—Mo1—Mo1^x^	60.849 (16)	Mo2^ix^—Mo3—Mo3^viii^	89.04 (2)
Mo1^xiii^—Mo1—Mo1^xiv^	90	Mo2^ix^—Mo3—Mo3^ix^	60.094 (17)
Mo1^x^—Mo1—Mo1^xiv^	58.302 (17)	Mo2^ix^—Mo3—Mo3^xv^	110.275 (17)
Mo2^viii^—Mo2—Mo2^ix^	60.00 (2)	Mo2^ix^—Mo3—Mo3^vi^	145.90 (3)
Mo2^viii^—Mo2—Mo3	90.953 (18)	Mo3^viii^—Mo3—Mo3^ix^	60.000 (18)
Mo2^viii^—Mo2—Mo3^viii^	60.546 (16)	Mo3^viii^—Mo3—Mo3^xv^	90
Mo2^ix^—Mo2—Mo3	62.472 (16)	Mo3^viii^—Mo3—Mo3^vi^	60.02 (2)
Mo2^ix^—Mo2—Mo3^viii^	89.850 (19)	Mo3^ix^—Mo3—Mo3^xv^	60.019 (16)
Mo3—Mo2—Mo3^viii^	57.92 (2)	Mo3^ix^—Mo3—Mo3^vi^	90
Mo2—Mo3—Mo2^ix^	56.983 (15)	Mo3^xv^—Mo3—Mo3^vi^	59.962 (18)
Mo2—Mo3—Mo3^viii^	61.982 (19)		

Symmetry codes: (i) −*x*+1/3, −*y*+2/3, −*z*+2/3; (ii) −*y*+1, *x*−*y*+1, *z*; (iii) −*x*+*y*, −*x*+1, *z*; (iv) −*x*+1, −*y*+1, −*z*+1; (v) *y*, −*x*+*y*+1, −*z*+1; (vi) *x*−*y*, *x*, −*z*+1; (vii) −*x*, −*y*+1, −*z*+1; (viii) −*y*, *x*−*y*, *z*; (ix) −*x*+*y*, −*x*, *z*; (x) *y*−2/3, −*x*+*y*−1/3, −*z*+2/3; (xi) *x*−*y*+1/3, *x*−1/3, −*z*+2/3; (xii) −*y*, *x*−*y*+1, *z*; (xiii) −*x*+*y*−1, −*x*, *z*; (xiv) *x*−*y*+1/3, *x*+2/3, −*z*+2/3; (xv) *y*, −*x*+*y*, −*z*+1.

## References

[bb1] Altomare, A., Burla, M. C., Camalli, M., Cascarano, G. L., Giacovazzo, C., Guagliardi, A., Moliterni, A. G. G., Polidori, G. & Spagna, R. (1999). *J. Appl. Cryst.* **32**, 115–119.

[bb2] Becker, P. J. & Coppens, P. (1974). *Acta Cryst.* A**30**, 129–147.

[bb3] Brandenburg, K. (2001). *DIAMOND* Crystal Impact GbR, Bonn, Germany.

[bb4] Duisenberg, A. J. M., Kroon-Batenburg, L. M. J. & Schreurs, A. M. M. (2003). *J. Appl. Cryst.* **36**, 220–229.

[bb5] Nonius (1998). *COLLECT* Nonius BV, Delft, The Netherlands.

[bb6] Petříček, V., Dušek, M. & Palatinus, L. (2006). *JANA2006* Institute of Physics, Praha, Czech Republic.

[bb7] Potel, M., Chevrel, R. & Sergent, M. (1980). *Acta Cryst.* B**36**, 1319–1322.

[bb8] Schäfer, H. & von Schnering, H. G. (1964). *Angew. Chem.* **76**, 833–845.

[bb9] Shannon, R. D. (1976). *Acta Cryst.* A**32**, 751–767.

[bb10] Spek, A. L. (2009). *Acta Cryst.* D**65**, 148–155. 10.1107/S090744490804362X PMC2631630 19171970

